# Risk factors for suicide in prisons: a systematic review and meta-analysis

**DOI:** 10.1016/S2468-2667(20)30233-4

**Published:** 2021-02-10

**Authors:** Shaoling Zhong, Morwenna Senior, Rongqin Yu, Amanda Perry, Keith Hawton, Jenny Shaw, Seena Fazel

**Affiliations:** a Department of Psychiatry and National Clinical Research Center for Mental Disorders, The Second Xiangya Hospital, Central South University, Changsha, China; b Department of Psychiatry, University of Oxford, Oxford, UK; c Department of Health Sciences, University of York, York, UK; d Centre for Suicide Research, Department of Psychiatry, University of Oxford, Oxford, UK; e Oxford Health NHS Foundation Trust, Warneford Hospital, Oxford, UK; f University of Manchester, Greater Manchester Mental Health Trust, Manchester, UK

## Abstract

**Background:**

Rates of suicide among people in prison are elevated compared with people of similar age and sex who are living in the community. Improving assessments and interventions to reduce suicide risk requires updated evidence on risk factors. We aimed to examine risk factors associated with suicide in prisoners.

**Methods:**

We did an updated systematic review and meta-analysis of risk factors for suicide among people in prison. We searched five biblographic databases for articles published between Jan 1, 2006, and Aug 13, 2020, and one database for articles published between Jan 1, 1973, and Aug 13, 2020. Eligible studies reported risk factors in individuals who died by suicide while in prison and in controls from the general prison population. Two reviewers independently extracted data for each study using a standardised form. We calculated random-effects pooled odds ratios (ORs) for the association of suicide with demographical, clinical, criminological, and institutional risk factors, and investigated heterogeneity using subgroup and meta-regression analyses. This systematic review is registered with PROSPERO, CRD42020137979.

**Findings:**

We identified 8041 records through our searches, and used 77 eligible studies from 27 countries, including 35 351 suicides, in the main analysis. The strongest clinical factors associated with suicide were suicidal ideation during the current period in prison (OR 15·2, 95% CI 8·5–27·0), a history of attempted suicide (OR 8·2, 4·4–15·3), and current psychiatric diagnosis (OR 6·4, 3·6–11·1). Institutional factors associated with suicide included occupation of a single cell (OR 6·8, 2·3–19·8) and having no social visits (OR 1·9, 1·5–2·4). Criminological factors included remand status (OR 3·6, 3·1–4·1), serving a life sentence (OR 2·4, 1·3–4·6), and being convicted of a violent offence, in particular homicide (OR 3·1, 2·2–4·2).

**Interpretation:**

Several modifiable risk factors, such as psychiatric diagnosis, suicidal ideation during the current period in prison, and single-cell occupancy, are associated with suicide among people in prison. Preventive interventions should target these risk factors and include improved access to evidence-based mental health care. Understanding other factors associated with suicide might improve risk stratification and resource allocation in prison services.

**Funding:**

Wellcome Trust, National Institute for Health Research Applied Research Collaboration Oxford and Thames Valley.

## Introduction

Deaths by suicide among people in prison have long been shown to occur at higher rates than among general populations of similar ages. In a study done in 24 high-income countries in 2013–17, suicide rates in male prisoners were 3–8 times higher than in the general population, whereas the rate in female prisoners was typically more than 10 times higher.^1^ Approaches to reduce suicide risk in prisons include risk assessment and management for individual prisoners, and targeting modifiable risk factors.^2^, ^3^

A previous systematic review suggested that some modifiable environmental and clinical factors were associated with suicide in prison, but for other potentially important risk factors the existing evidence was insufficient to confidently assess their association with suicide.^4^ The search done in this previous review^4^ only used articles published until 2007. Since that time, several new studies have been done, particularly on the contribution of mental health. In addition, prison populations have increased in size in many countries, and this increase could have altered the contribution of risk factors.

We did a systematic review and meta-analysis to update the evidence on suicide in prison, investigate new associations, and improve the precision of estimated effect sizes of previously identified risk factors. We aimed to provide a quantitative synthesis of evidence from case-control and case-cohort studies comparing prisoners who died by suicide with those who did not.

## Method

### Study designs and participants

We followed the Preferred Reporting Items for Systematic Reviews and Meta-Analysis guidelines^5^ and the Meta-analyses of Observational Studies in Epidemiology proposal.^6^

**Research in context**

**Evidence before this study**

One previous systematic review has synthesised evidence on risk factors associated with suicide in prisons, but its search for relevant publications ended in 2007. Since this review, several studies have been published and worldwide prison populations have increased, with the likelihood that prisoners with different background risks are now entering prisons. To identify other reviews on prison suicide, we searched Embase, MEDLINE, PsycINFO, CINAHL, and Global Health without language restrictions for papers published between Jan 1, 2006, and Aug 13, 2020. We used a combination of search terms related to suicide (ie, “suicid*”) and people in prison (“prison*” OR “felon*” OR “detain*” OR “jail or custod*” OR “[her majesty's prison]” OR “remand*” OR “offender*” OR “institution” OR “panel” OR “inmate*” OR “correction*” OR “sentenced” OR “incarcerat*” OR “gaol*”). We did not identify any other systematic reviews that quantitatively examined risk factors for suicide in prisoners. One narrative review summarised studies of near-lethal suicide attempts in prison and outlined potential intervention strategies.

**Added value of this study**

In this systematic review and meta-analysis of 77 studies, we provide an updated synthesis of the range and magnitude of risk factors associated with suicide in prisons. This review provided more precise results than previous work, and clarified the direction of effects for several factors for which there was uncertainty. Previous suicidal attempts, psychiatric diagnosis, occupation of a single cell, absence of social visits, and alcohol misuse were all associated with suicide. Moreover, being convicted of a sexual offence was associated with a higher risk of suicide than other offence types, which might inform risk assessment on arrival to prison.

**Implications of all the available evidence**

Preventive interventions should target potentially modifiable risk factors, such as the identification and treatment of mental health problems and alcohol misuse. Many factors are associated with small relative risks; therefore, suicide risk assessment should combine multiple risk factors with appropriate weighting and be informed by clinical decision making. Universal interventions will be an important component of suicide prevention strategies in light of challenges involved in predicting individual risk. Future research could examine how risk factors differ by age and sex, and whether prediction modelling can improve assessment of suicide risk.

### Search strategy and selection criteria

For this systematic review and meta-analysis, we searched five bibliographic databases, namely, Embase (from Jan 1, 2006, to Aug 13, 2020), MEDLINE (from Jan 1, 2006, to Aug 13, 2020), PsycINFO (from Jan 1, 2006, to Aug 13, 2020), CINAHL (from Jan 1, 2006, to Aug 13, 2020), and Global Health (from database inception [Jan 1, 1973] to Aug 13, 2020). For this update, we used the same strategy as for the previous systematic review:^4^ we used a combination of two search terms: suicide (ie, “suicid*”) and prisoners (“prison*” OR “felon*” OR “detain*” OR “jail” OR “custod*” OR “[her majesty's prison]” OR “remand*” OR “offender*” OR “institution” OR “panel” OR “inmate*” OR “correction*” OR “sentenced” OR “incarcerat*” OR “gaol*”), and we scanned the bibliographies of articles included in the updated review and of studies cited in the previous review. We also searched for grey literature (eg, reports, government documents, dissertations, theses, conference abstracts) using Google Scholar (see appendix pp 1–5 for full search strategies and results). Relevant study authors were contacted when additional data or clarifications were required. Non-English surveys were translated.

### Eligibility assessment

We included studies from all countries and in all languages. Studies were included when they were quantitative studies that identified risk factors for suicide in people in prison compared with matched or randomly selected controls or the total or average prison population, and when absolute numbers of suicide deaths were provided or could be extracted from the data provided. We excluded investigations in selected samples (eg, individuals with mental disorders or drug users) and selected outcomes (eg, hanging) as well as studies that compared prisoners who died by suicide with the general population, and those with another outcome of suicide risk rather than death by suicide (eg, outcomes of deliberate self-harm or attempted suicide). We also excluded case reports, case series, reviews, and qualitative studies. For studies reporting on a comprehensive sample of suicide deaths without a control group, we searched for information on the general prison population for the same variables from government reports for a similar time period and used these data as control data for the corresponding groups. A primary study was excluded if a control group could not be identified. To avoid duplication of samples, we included the study with the largest sample or with the longest follow-up period when cases came from overlapping populations. Two researchers (SZ and MS) did the initial screening of abstracts and full-text publications for eligibility. Any uncertainties between the two researchers were discussed with a third author (RY) to reach consensus.

### Design of studies and data extraction

A standardised form was used to independently extract data on geographical location, study design, the period of study, the definition of suicide (suicide only, suicide and open verdicts, or not reported), the absolute number of suicide cases, average age, and sex. Two reviewers (SZ and MS) independently extracted data for each study, and reached consensus on inconsistencies by discussion between them and with RY. For suicide cases and controls, we extracted data on demographic, criminological, and clinical variables examined in the previous review.^4^ For criminological variables, offence categories included people convicted and charged. Homicide includes murder and manslaughter.

We classified included studies into two groups by the type of control group: group 1 studies compared people who had died from suicide in prison with people from a randomly selected or matched control group from the same prison population, whereas group 2 studies compared characteristics of prisoners who died of suicide with characteristics of the total or average prison population during a matched period. Similar to the original review, we calculated the sample size of the control group to be proportional to that of the case group to avoid bias.

To assess risk of bias, we considered using the Newcastle-Ottawa Quality Assessment Scale. However, two items—the ascertainment of exposure and non-response rate—were not applicable and did not vary across studies. Thus, we used the OHAT (Office of Health Assessment and Translation) tool, which can be applied to case-control studies and cross-sectional studies. The mean quality score of the included case-control studies was around 5 on a 9 point scale (range 4–6), indicating overall medium quality. All case-control studies used control groups from the same prison. In addition, all studies used the same methods to ascertain risk factors for suicide cases and controls. However, all studies used prison or medical records for information on risk factors, which introduces a higher risk of bias than other data sources such as structured interviews.

### Statistical analysis

There were no deviations from the review protocol. We combined included studies from this update with the original review for analysis. For a given risk factor, we combined number of cases with the risk factor and the total number of participants in each study for both case and control group. We generated pooled odds ratios (ORs) with 95% CIs for risk factors reported in two or more studies using random-effects models. In the analyses, we excluded studies when the explored risk factors (eg, age, sex, sentence) were matched in the control group for group 1 studies. We investigated sources of heterogeneity using Cochran Q and the *I*^2^ statistics. *I*^2^ is reported as a percentage out of 100%, whereby 0–40% denotes that the heterogeneity might not be important, 30–60% might present moderate heterogeneity, 50–90% refers to substantial heterogeneity, and 75–100% indicates considerable heterogeneity.^7^ We did subgroup analyses to examine whether there were differences in outcomes on the basis of study design (group 1 *vs* group 2), type of publication (peer-reviewed paper *vs* grey literature), and country (USA *vs* other countries). For the heterogeneity analysis, we examined risk factors that were included in more than ten studies, and then looked at the heterogeneity between the studies reporting the risk factor—thus, for the following variables: sex, race or ethnicity, marital status, age, detainee or remand status, and type of offence.^8^ Furthermore, we did meta-regression for those risk factors that showed considerable heterogeneity (≥75%). All analyses were done using R (version 3.6.0)^9^ and R package meta (version 4.9–9).^10^ The review is registered with PROSPERO, CRD42020137979.

### Role of the funding source

The funder of the study had no role in study design, data collection, data analysis, data interpretation, or writing of the report. All authors had full access to all the data in the study and had final responsibility for the decision to submit for publication.

## Results

**Figure 1:**
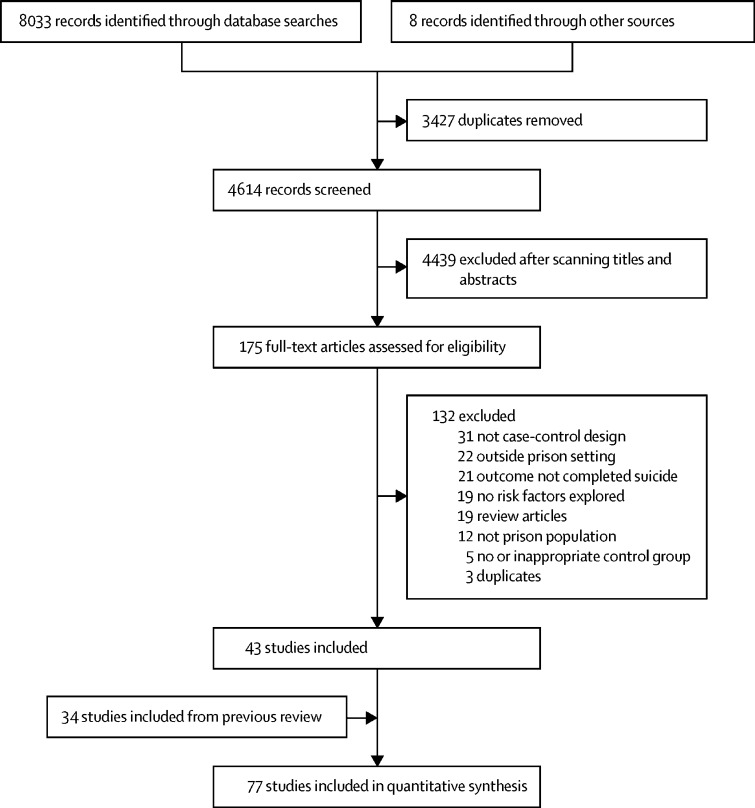
Study selection

We identified 8033 relevant studies in the database search and eight additional records through other sources, of which 43 new studies, not indentified in the 2007 systematic review,^4^ met eligibility criteria (figure 1).^1^, ^11^, ^12^, ^13^, ^14^, ^15^, ^16^, ^17^, ^18^, ^19^, ^20^, ^21^, ^22^, ^23^, ^24^, ^25^, ^26^, ^27^, ^28^, ^29^, ^30^, ^31^, ^32^, ^33^, ^34^, ^35^, ^36^, ^37^, ^38^, ^39^, ^40^, ^41^, ^42^, ^43^, ^44^, ^45^, ^46^, ^47^, ^48^, ^49^, ^50^, ^51^, ^52^ The total number of identified publications for the meta-analysis was 77, of which 34 were from the original review^53^, ^54^, ^55^, ^56^, ^57^, ^58^, ^59^, ^60^, ^61^, ^62^, ^63^, ^64^, ^65^, ^66^, ^67^, ^68^, ^69^, ^70^, ^71^, ^72^, ^73^, ^74^, ^75^, ^76^, ^77^, ^78^, ^79^, ^80^, ^81^, ^82^, ^83^, ^84^, ^85^, ^86^ (see appendix pp 7–13 for characteristics of studies). Across the 77 included studies, the total number of prisoner suicide deaths was 35 351.

The 77 included studies were done with data from 27 different countries: 28 investigations (n=14 650 cases or 41·4% of prison suicide deaths) from the USA, 14 (n=4854 cases, 13·7%) from England and Wales, eight (n=3465 cases, 9·8%) from Germany, four (n=210 cases, 0·6%) from Australia, four (n=1487, 4·2%) from Italy, three (n=556 cases, 1·6%) from France, three (n=224 cases, 0·6%) from Scotland, and two (n=101 cases, 0·3%) from Canada; furthermore, nine investigations (n=5880, 16·6%) were based in other high-income countries (Austria, Belgium, Denmark, Iceland, the Netherlands, New Zealand, Norway, Spain, Switzerland). One study included people in prison from 24 countries (n=2810, 7·5%) in Europe, another included people in prison from 10 countries (n=1324, 3·7%) in South America.

Several static and dynamic risk factors reported in more than one study are presented in Table 1, Table 2 (and factors reported in solely one study are presented in the appendix pp 14–15).

**Table 1:** Static risk factors associated with suicide in prisoners (in domains and ordered by odds ratio)

	Study group*	**Number of studies**	**Cases, n/N (%)**†	**Controls, n/N (%)**†	**Pooled odds ratio (95% CI)**	**z**	**p value**	***I*^2^, %**
**Demographics**								
Homeless^58^, ^81^, ^84^, ^85^	1 and 2	4	55/318 (10·4%)	104/851 (12·2%)	2·4 (0·3–19·8)	0·8	0·43	94%
White^12^, ^13^, ^19^, ^28^, ^29^, ^40^, ^54^, ^55^, ^58^, ^59^, ^62^, ^63^, ^64^, ^65^, ^66^, ^67^, ^68^, ^69^, ^73^, ^74^, ^81^, ^83^	1 and 2	22	8117/11 773 (68·9%)	5766/12112 (47·6%)	2·0 (1·4–2·7)	4·2	<0·0001	95%
Married^12^, ^54^, ^58^, ^59^, ^60^, ^75^, ^76^, ^78^, ^81^, ^83^	1 and 2	10	518/1800 (28·8%)	500/2263 (22·1%)	1·5 (1·2–1·7)	5·2	<0·0001	0%
Employed^12^, ^58^, ^60^, ^78^, ^81^	1	5	317/673 (47·1%)	521/1224 (42·6%)	1·3 (0·5–3·2)	0·5	0·59	95%
Male sex^1^, ^11^, ^15^, ^17^, ^19^, ^21^, ^22^, ^24^, ^25^, ^27^, ^28^, ^29^, ^33^, ^38^, ^41^, ^46^, ^47^, ^48^, ^50^, ^51^, ^54^, ^55^, ^56^, ^57^, ^63^, ^65^, ^66^, ^67^, ^68^, ^69^, ^73^, ^75^, ^76^, ^80^, ^81^, ^82^, ^83^	1 and 2	37	18 819/20 080 (93·7%)	18 616/20 263 (91·9%)	1·2 (1·0–1·5)	2·1	0·036	60%
Older than 50 years^13^, ^19^, ^37^, ^45^, ^47^, ^61^, ^64^, ^75^, ^76^, ^80^	2	10	635/4736 (13·4%)	535/4736 (11·3%)	1·2 (1·0–1·4)	2·4	0·017	23%
Aged 31–40 years^13^, ^19^, ^24^, ^26^, ^37^, ^61^, ^64^, ^75^, ^76^, ^80^	2	10	767/2448 (31·3%)	716/2448 (29·2%)	1·1 (1·0–1·2)	1·5	0·12	8%
Aged 41–50 years^13^, ^19^, ^26^, ^37^, ^61^, ^64^, ^75^, ^76^, ^80^	2	9	425/2370 (17·9%)	389/2370 (16·4%)	1·1 (0·9–1·3)	0·5	0·59	36%
Education not continued after age 16 years^60^, ^76^, ^81^	1 and 2	3	235/449 (52·3%)	430/850 (50·6%)	0·9 (0·4–2·4)	−0·2	0·87	91%
Aged 21–30 years^26^, ^40^, ^61^, ^75^, ^76^, ^80^	2	6	402/1075 (37·4%)	423/1075 (39·3%)	0·9 (0·8–1·2)	−0·6	0·56	19%
Aged 16–20 years^61^, ^67^, ^75^, ^76^, ^80^	2	5	91/617 (14·7%)	103/617 (16·7%)	0·8 (0·6–1·2)	−1·0	0·32	8%
Aged 18–29 years^13^, ^19^, ^37^, ^41^, ^50^	2	5	726/2279 (31·9%)	868/2279 (38·1%)	0·7 (0·7–0·8)	−4·6	<0·0001	0%
Hispanic^28^, ^29^, ^59^, ^62^, ^63^, ^66^	2	6	1200/8694 (13·8%)	1996/8694 (23·0%)	0·6 (0·5–0·8)	−3·8	<0·0001	68%
Black^13^, ^14^, ^19^, ^21^, ^28^, ^29^, ^54^, ^59^, ^62^, ^63^, ^64^, ^66^, ^68^, ^69^, ^73^, ^74^, ^83^	1 and 2	17	1786/11 895 (15·0%)	4551/11 901 (38·2%)	0·4 (0·3–0·5)	−7·8	<0·0001	87%
**Criminal history**								
Detainee or remand status^11^, ^12^, ^16^, ^19^, ^24^, ^28^, ^31^, ^35^, ^40^, ^46^, ^48^, ^50^, ^56^, ^57^, ^71^, ^73^, ^74^, ^75^, ^76^, ^77^, ^79^, ^81^, ^83^	1 and 2	23	6467/9722 (66·5%)	4242/9918 (42·8%)	3·6 (3·1–4·1)	17·1	<0·0001	61%
Homicide‡^11^, ^12^, ^20^, ^24^, ^30^, ^43^, ^50^, ^53^, ^55^, ^57^, ^59^, ^65^, ^68^, ^76^, ^78^, ^80^, ^85^	1 and 2	17	649/3427 (18·9%)	255/3599 (7·1%)	3·1 (2·2–4·2)	6·8	<0·0001	68%
Life sentence^12^, ^20^, ^47^, ^57^, ^68^, ^73^, ^74^, ^76^, ^80^	1 and 2	9	225/1970 (11·4%)	107/1975 (5·4%)	2·4 (1·3–4·6)	2·7	0·0066	77%
Violent offence‡^12^, ^20^, ^30^, ^43^, ^50^, ^57^, ^58^, ^68^, ^76^, ^80^	1 and 2	10	611/2913 (21·0%)	410/3065 (13·4%)	2·1 (1·4–3·0)	3·7	0·0002	83%
Length of sentence ≥18 months but not life sentence^20^, ^56^, ^57^, ^68^, ^80^	2	5	226/421 (53·7%)	174/421 (41·3%)	1·5 (0·9–2·4)	1·7	0·090	60%
Previous conviction^12^, ^58^, ^60^, ^81^, ^83^	1 and 2	5	519/735 (70·6%)	743/1127 (65·9%)	1·5 (0·8–3·0)	1·3	0·20	89%
Sexual offence‡^13^, ^19^, ^20^, ^24^, ^30^, ^43^, ^47^, ^50^, ^57^, ^68^, ^72^, ^74^, ^76^, ^80^	2	14	627/5570 (11·3%)	481/5570 (8·6%)	1·4 (1·1–1·9)	2·7	0·0062	69%
Burglary, robbery, or theft offence‡^13^, ^19^, ^20^, ^24^, ^28^, ^30^, ^43^, ^57^, ^68^, ^74^, ^76^	2	11	1771/7699 (23·0%)	2099/7517 (27·9%)	0·7 (0·6–0·9)	−2·7	0·0063	78%
Length of sentence <18 months‡^56^, ^57^, ^68^, ^80^	2	4	77/300 (25·7%)	148/300 (49·3%)	0·4 (0·2–0·9)	−2·1	0·035	78%
Drug offence‡^13^, ^19^, ^20^, ^28^, ^30^, ^43^	2	6	669/6929 (9·7%)	1524/6859 (22·2%)	0·4 (0·3–0·5)	−6·7	<0·0001	71%
Sentenced status^11^, ^19^, ^32^, ^46^, ^56^, ^57^, ^73^, ^77^, ^79^	2	9	1419/2782 (51·0%)	2225/2782 (80·0%)	0·3 (0·2–0·4)	−7·9	<0·0001	82%

Risk factors were compared with other people in prison.

* Group 1 studies use a randomly selected or matched control group and group 2 studies use the total or average prison population during a matched period.

† n denotes number of cases or controls with risk factor and N denotes total number of cases or controls.

‡ Explored for people on remand and for people sentenced.

**Table 2:** Dynamic risk factors associated with suicide in prisoners (in domains and ordered by odds ratio)

	Study group*	**Number of studies**	**Cases, n/N (%)**†	**Controls, n/N (%)**†	**Pooled odds ratio (95% CI)**	**z**	**p value**	***I*^2^, %**
**Clinical risk factors**								
Suicidal ideation^60^, ^84^	1	2	93/250 (37·2%)	15/401 (3·7%)	15·2 (8·5–27·0)	9·2	<0·0001	0%
History of attempted suicide^58^, ^60^, ^83^, ^84^	1	4	198/400 (49·5%)	81/753 (10·8%)	8·2 (4·4–15·3)	6·7	<0·0001	63%
History of self-harm^12^, ^58^, ^60^, ^83^, ^84^	1 and 2	5	312/611 (51·1%)	123/970 (12·7%)	7·1 (4·4–11·5)	7·9	<0·0001	65%
Current psychiatric diagnosis^12^, ^20^, ^36^, ^58^, ^60^, ^83^, ^84^	1 and 2	7	341/791 (43·1%)	115/985 (11·7%)	6·4 (3·6–11·1)	6·5	<0·0001	75%
Depression^36^, ^65^	1 and 2	2	17/62 (27·4%)	5/62 (8·1%)	4·9 (1·6–14·8)	2·8	0·0052	0%
Psychotropic medication^12^, ^60^, ^78^	1	3	187/483 (38·7%)	93/654 (14·2%)	3·8 (2·8–5·1)	9·1	<0·0001	0%
Alcohol misuse^12^, ^58^, ^83^	1	3	140/378 (37·0%)	106/560 (18·9%)	2·5 (1·4–4·3)	3·2	0·0014	68%
Poor physical health^65^, ^83^	1	2	31/86 (36·0%)	32/125 (25·6%)	2·0 (0·7–5·9)	1·3	0·10	26%
**Institutional risk factors**								
Single-cell occupancy^12^, ^60^, ^65^	1 and 2	3	310/435 (71·3%)	167/519 (32·2%)	6·8 (2·3–19·8)	3·5	0·0004	90%
No social visits^12^, ^50^, ^60^	1 and 2	3	445/750 (59·3%)	426/907 (47·0%)	1·9 (1·5–2·4)	4·3	<0·0001	20%

* Group 1 studies use a randomly selected or matched control group and group 2 studies use the total or average prison population during a matched period.

† n denotes number of cases or controls with risk factor and N denotes total number of cases or controls.

We first examined demographic factors (table 1; appendix p 6). Factors most strongly associated with suicide risk included white race or ethnicity (OR 2·0, 95% CI 1·4–2·7; but there was substantial heterogeneity between studies (*I*^2^=95%), being married (OR 1·5, 1·2–1·7), and male sex (OR 1·2, 1·0–1·5; table 1). In addition, in the ten studies that investigated nationality, not being a citizen of the country of incarceration was inversely linked to suicide risk (OR 0·7, 0·6–1·0, p=0·02; appendix p 14). There were no clear associations with age among the groups older than 25 years (OR 1·2, 0·9–1·7), older than 30 years (OR 1·3, 0·8–1·9), or older than 45 years (OR 0·8, 0·6–1·1). There was no clear association with having no formal education beyond age 16 years (OR 0·9, 0·4–2·4; appendix p 6).

**Figure 2:**
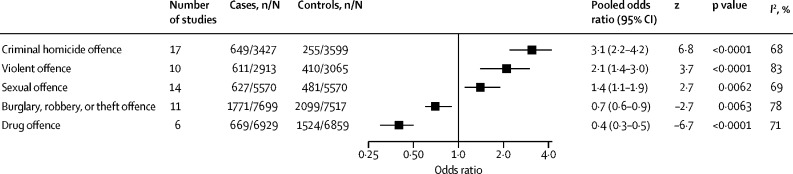
Risk of suicide in people in prison based on most recent offence category In remand prisoners, alleged offences were examined. n=number of cases or controls with risk factor. N=total number of cases or controls.

With respect to criminological factors, the following were associated with increased risk of prison suicide: being a detainee or on remand (OR 3·6, 95% CI 3·1–4·1) and serving a life sentence (OR 2·4, 1·3–4·6). In relation to offence categories, being convicted of criminal homicide (OR 3·1, 2·2–4·2) and sexual offences (OR 1·4, 1·1–1·9) were associated with an increased risk of suicide (table 1; figure 2). In addition, violent offences (excluding homicide and sexual offences) were also associated with suicide (OR 2·1, 1·4–3·0), but there was substantial heterogeneity between studies (*I*^2^=83%). Conversely, conviction for a drug offence showed an inverse association with suicide (OR 0·4, 0·3–0·5). Being sentenced was associated with a reduced suicide risk (OR 0·3, 0·2–0·4) when compared with detainee or remand status.

**Figure 3:**
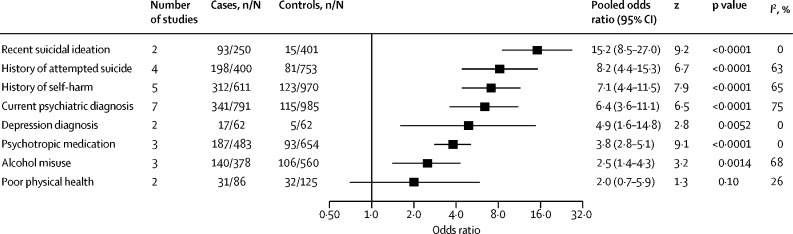
Risk of suicide in people in prison based on clinical factors n=number of cases or controls with risk factor. N=total number of cases or controls.

Clinical factors associated with suicide were suicidal ideation during their current period in prison (OR 15·2, 8·5–27·0), having a history of attempted suicide (OR 8·2, 4·4–15·3), having a history of self-harm (OR 7·1, 4·4–11·5), and being prescribed psychotropic medication (OR 3·8, 2·8–5·1; table 2; figure 3). Regarding individual disorders, a current psychiatric diagnosis (OR 6·4, 3·6–11·1), a depression diagnosis (OR 4·9, 1·6–14·8), and alcohol misuse (OR 2·5, 1·4–4·3) were each associated with increased risk. Poor physical health was not significantly associated with suicide (OR 2·0, 0·7–5·9), although this could be due to the small sample available to assess this factor. The heterogeneity for clinical factors ranged from 0% to 75%.

We examined institutional factors and found that occupation of a single cell (OR 6·8, 95% CI 2·3–19·8) and having no social visits (OR 1·9, 1·5–2·4) were associated with an increased risk of suicide (table 2; appendix p 6). However, there was substantial heterogeneity between studies.

We examined sources of heterogeneity by studying four possible explanations for the differences in the effects of risk factors between studies: country group, study design, type of publication, and sex. On meta-regression, for the variable of age older than 50 years, some heterogeneity was explained by differences between studies from the USA (OR 1·4, 95% CI 1·2–1·6) and other countries (OR 1·0, 0·9–1·2; Q=5·0, df=1, p=0·003; data not shown). No clear differences by country group were found in the associations between other risk factors and suicides. We found no differences in ORs by study design (group 1 *vs* group 2). In relation to sex, there were only two studies that examined risk factors specifically in female prisoners.^34^, ^40^ A study from England and Wales examined risk factors for suicide among female prisoners with a control group of female prisoners. The study reported associations between suicide and remand status (OR 3·0, 1·5–5·9), suicide and violent offending (OR 2·4, 1·2–5·2), and suicide and white ethnicity (OR 3·1, 1·4–7·3).^40^ Associations by age groups in women in prison were not clear, and there was only one suicide case in prisoners aged 50 years or older in this study population.^40^ In another study including 30 female prisoners who died by suicide in Germany, seven women had a psychiatric diagnosis, eight had a previous suicide attempt, and seven had shown evidence of drug withdrawal.^32^ Information on these risk factors was not reported from a female control group, but when compared with men who died by suicide in prison, drug withdrawal was more common for women who died from suicide in prison (27% *vs* 10%, p=0·01), whereas rates were similar for psychiatric disorder (27% for women *vs* 20% for men) and history of suicide attempt (33% for women *vs* 26% for men). Because 75 of 77 included studies did not report adjusted ORs, we presented the differences between adjusted and crude OR on risk factors (appendix p 18).

## Discussion

This updated systematic review and meta-analysis was based on 35 351 suicides among people in prison from 77 studies in 27 countries, and synthesised risk factors for suicide by clinical, criminological, demographic, and institutional domains. The five strongest factors associated with suicide risk were suicidal ideation during current period in prison, previous suicide attempt, history of self-harm, single-cell occupancy, and current psychiatric diagnosis. Our results suggest that several criminological variables are also associated with suicide risk, including remand status and offence type, particularly homicide.

This update provides new evidence in three ways. First, it adds precision to the associations reported in the previous systematic reviews,^4^, ^87^, ^88^ which is important for modifiable risk factors that can guide the development of preventive interventions. Second, it provides new data on two factors—depression diagnosis and absence of social visits—that were not identified in the previous reviews. Third, for some risk factors for which there was uncertainty, this update has clarified the direction of effects. Specifically, we have shown that an index sexual offence is associated with increased risk. We identified only one new case-control study published since 2006. The paucity of recent research is notable and suggests that facilitating prison research should be a central part of any strategy to reduce deaths in custody.

Our results highlight potentially modifiable risk factors that can be targeted by interventions as part of prevention efforts. The importance of recognition and treatment of mental health problems among prisoners is underscored by this review,^88^ and the strong associations reported should be considered in health-care service development and prison policy. Mental health services do not only need to be universally available to people in prison, but also adequately resourced and linked to effective interventions to address the higher prevalence of mental health diagnoses among prisoners than community-residing peers.^39^, ^89^, ^90^ Many countries screen individuals for mental health problems on arrival in prison.^91^ People identified as at risk of suicide should be assessed promptly by a mental health professional,^91^ and access to mental health services during incarceration should be similar to what is available for the general population.^92^ These services should consider access to psychological therapies with an evidence base in prisons and other settings.^93^, ^94^ However, despite these recommended standards, access to mental health care for people in prison is inconsistent and frequently delayed.^94^ Unmet mental health needs are likely to contribute to high rates of suicide among prisoners. Alongside provision of mental health care, prison staff require adequate training in recognising and responding to self-harm and other mental health needs to improve access to appropriate care.^95^, ^96^

Apart from clinical risk factors, we report associations between suicide and some modifiable institutional risk factors. One identified factor is absence of social visits.^12^, ^42^ Absence of visits might reflect a poor supportive social network, consistent with findings of previous work showing that male prisoners who have been involved in near-lethal suicide attempts have reduced social support compared with controls.^97^ This difference might reflect a complex combination of psychosocial needs for some prisoners, for whom pre-existing impulsivity and aggression could act as a shared risk factor for suicidality, criminal behaviour, and lack of social connections. On the other hand, it is possible that prison policies contribute to a lack of social visits, such as restrictive visiting practices or locating prisoners far from their homes. If this is the case, reducing such practices might contribute to suicide prevention. Ensuring families and friends can visit regularly could involve more third-sector organisations. Addressing issues of social connectedness requires a context-specific approach for people in prison. An example is the increased risk of suicide in married prisoners,^12^, ^52^ which contrasts with findings in the general population.^98^ Another institutional risk factor associated with suicide was being in a single cell. This finding highlights the importance of careful risk planning for all prisoners who express suicidal thoughts. Furthermore, this finding might be driven by practices—eg, individuals at higher risk (including those presenting with acute mental health problems) are more likely to be placed in single cells than lower risk prisoners.

Several non-modifiable risk factors, such as offence type and ethnicity, were also found to be associated with suicide. Previous studies have highlighted a positive association between white ethnicity and suicide in prison, a finding that was supported in our meta-analysis.^13^, ^52^ This association is likely to be driven by background differences in suicide rates observed in the general population,^99^ and might also be affected by confounding factors in some countries, including age and length of imprisonment.^12^, ^13^

Another implication of these findings is that non-modifiable risk factors, such as ethnicity and offence type, might assist suicide prevention by facilitating identification of individuals at high risk using structured instruments that incorporate these factors. Screening for suicide risk, despite being recommended in many jurisdictions,^91^, ^96^ tends to be based on one or two questions during a wider health-care assessment on arrival to prison.^100^, ^101^ When structured tools are used, they do not necessarily incorporate multiple weighted risk factors based on sufficiently large samples and often have not been subject to appropriate external validation.^102^ Future research needs to investigate whether stratification of risk can be accurately done. If so, safety planning can be supplemented with more resource-intensive suicide prevention therapies. Single risk factors are not sufficient to identify individuals at high risk of suicide. We estimated the positive predictive value (PPV) of the risk factors identified in this review, applied to a prison population with an annual suicide rate of 83 deaths per 100 000 prisoners (ie, the average rate for England and Wales, UK, from 2011 to 2014).^19^ The PPV of any particular single risk factor is low, for example current psychiatric diagnosis had a PPV of 0·3% and single cell accommodation a PPV of 0·2%. Combining two risk factors can increase predictive performance—combining current psychiatric diagnosis and single-cell accommodation gave a PPV of 0·7% (assuming risk factors are independent). It is unclear whether this predictive performance represents a clinically meaningful level of accuracy: the PPV is low, but could be useful in the context of a lower baseline prevalence of the outcome (which is less than 0·1% in England and Wales, UK, for example).

To maximise PPVs, screening and risk prediction tools should incorporate many risk factors with appropriate weighting, and it might be that such risk tools are used to screen out people at low risk. Even then, multiple weighted risk factors will be required and screening tools are likely to yield high numbers of false positive results. Considering the challenges involved in accurately assessing suicide risk, universal prevention strategies will continue to be an important complement to selective interventions. Examples of universal interventions include restriction of access to means, ensuring access to supportive social interactions, such as peer support programmes,^2^, ^103^ and promoting meaningful daytime activity.^104^

To assess the relevance of each potentially modifiable risk factor for suicide prevention, it is useful to consider both the effect size and prevalence of exposure in the prison population. The prevalence of rare risk factors (such as staying in a disciplinary cell [<1%]) might be too small to contribute to prevention initiatives. Among the controls, 47% had no social visits, 13% had a history of self-harm or suicide attempt, and 12% had a current psychiatric diagnosis. These findings suggest that a substantial proportion of the prison population is exposed to these modifiable risk factors, underscoring their importance as targets for preventive interventions.

Risk factors for suicide might differ between male and female prisoners, but most of the included studies combined data for both sexes, or only included male prisoners, with the exception of two studies.^32^, ^40^ One of these two studies showed similar associations between some non-modifiable factors across sexes, but differences in suicide risk by age groups were not clear.^40^ The other study found that a higher proportion of female rather than male prisoners who died by suicide had evidence of drug withdrawal.^32^ This difference might reflect higher levels of drug dependence on arrival to prison in female prisoners than in male prisoners.^105^ Differences in medical care or recognition of drug withdrawal in female prisoners might also contribute to this finding, which new research could investigate. Although the little available evidence suggests several risk factors are shared for both male and female prisoners, there is a need for future research to clarify differences by age and sex, and other risk factors, which could lead to more tailored assessment of risk, treatment allocation, and delivery of services.

One strength of the review is the large number of suicide cases (n=34 628). We identified 16 reports with 11 518 suicides (33% of all included suicides) from grey literature. There was a large number of suicides in group 2 studies, within which we identified 67 studies reporting 33 682 prison suicides (97% of all included suicides) that used the average or total prison population as the control group. The inclusion of group 2 studies has implications for interpretation. First, it is possible that the methods used to measure risk factors in prisoners who died by suicide differed from the methods used in control populations, which need to be considered in the 13 studies in which the control group data came from the general prison population. However, these studies tended to report variables such as offence type, remand status, or sex, which are reliable. Second, in these group 2 investigations, the control group included prisoners who have died by suicide, which means that the effect sizes will be more conservative. However, because suicide is a rare outcome, it is unlikely to have a large influence on effect sizes. Finally, combining case-control studies using matched controls with group 2 studies in which the control group was unmatched might have limited the precision of the pooled effect estimates for which matching was done. In other words, some group 1 studies did not contribute to risk estimates for selected socioeconomic and criminal history factors. Our decision to pool these group 2 investigations with case-control ones was supported by subgroup and meta-regression analyses, which found little evidence of differences in ORs based on study design. This approach has allowed us to combine data on a large number of suicide cases despite finding few case-control studies. Nonetheless, the analysis was underpowered for some risk factors such as level of education, for which only three studies were identified.

Several limitations should be considered. Definitions of suicide varied between studies, and it was not possible to test whether these discrepancies contributed to heterogeneity. For example, studies in England and Wales included all self-inflicted deaths as cases,^13^ whereas other studies included only suicides as determined by official medical reports,^28^ or included suicides and open verdicts.^26^ However, many studies did not report the criteria used to define suicide deaths so we were unable to examine whether differences in definitions were linked to effect sizes. In addition, there was insufficient information to examine risk factors by specific groups of prisoners (eg, those on remand) or by type of institution (eg, security level). Almost all studies did not adjust for potential confounds and we were therefore unable to account for the degree of bias that confounding could introduce in risk estimates for most studied risk factors. This lack of adjustment is a key gap in the evidence to date. The effect and implications of confounding will probably depend on the population studied and the analytic strategy used. For the two included reports for which we could investigate this issue, lack of adjustment for confounders resulted in overestimation of the effect of the studied risk factor (ranging from 7% to 85%). However, this comparison is limited by few relevant studies and does not account for possible interactions between confounds, particularly with age and sex. Future research should use multivariable models, which include sex,^36^, ^106^ age,^31^, ^107^ ethnicity,^106^ and remand status.^50^ In addition, unmeasured residual confounding from variables such as genetic factors and childhood adversity will probably contribute to bias in risk estimates. Therefore, future work should improve measurement of these possible confounds. Quasi-experimental methods, which partly account for these residual confounds, such as using family-based designs, could provide more evidence. For these designs, proxy outcomes (eg, self-harm) might be considered due to the low prevalence of suicide. Low statistical power for suicide outcomes will also be a challenge for trials, but trials could usefully examine service-related and institutional factors if cluster designs are considered. Furthermore, one possible limitation in our review is that we did not specifically search criminology databases, although one of the included databases (PsycINFO) did contain criminological and legal journals and we searched citations in included papers.

It is probable that there are additional individual and institutional risk factors for suicide in prison that were not studied in the included papers. Studies of prisoners who have made near-lethal suicide attempts have found an association with psychosocial factors such as past trauma, childhood abuse, and negative experiences of imprisonment including bullying.^97^, ^108^ Individual-level characteristics might interact with institutional factors, such as access to health services and aspects of staff–prisoner interaction. Incarceration rates and sentencing practices lead to heterogeneity in prison populations and as a result could affect the distribution of individual-level suicide risk factors. Countries with low incarceration rates are likely to have a higher proportion of people in prison for serious violent offences—with a potentially elevated suicide risk—compared with countries with high incarceration rates (which include prisoners convicted of non-violent offences, with lower risk for suicide).^1^ Findings on links with prison overcrowding have been inconsistent due to several influencing factors, including effects on staff–prisoner interactions and protective effects from double occupancy of single cells.^109^, ^110^

Future research should examine risk factor variation in low-income and middle-income countries. Some of the risk factors identified in this review, such as psychiatric diagnosis and substance use disorders, are highly prevalent among people in prison in low-income and middle-income countries.^89^ If there are differences in risk factors, they could inform the development of tailored prevention strategies.

The association between suicide and physical health problems still needs clarification in the prison context. Frequent transitions between, into, and out of criminal justice settings might complicate access to primary care, which could be addressed as one approach to improve the physical health of people in prisons. One other risk factor for which evidence was lacking is childhood adversity, which is common in prisoners.^111^ Future research could examine the links between childhood adversity, mental illness, substance use, and suicide in people in prison, as these factors frequently co-occur.^98^, ^99^

In conclusion, we have reported a range of demographic, criminological, clinical, and institutional risk factors associated with suicide in prisons. Our findings highlight modifiable risk factors, which could improve suicide prevention and intervention strategies. These strategies should in particular target those with previous suicidal behaviours, mental illness, and single-cell occupancy, and should include provision of psychological and pharmacological treatment for psychiatric disorders.

## Data sharing

Study data are available on request to the authors.
