# Supplementary material for: Risk factors for suicide in prisons: a systematic review and meta-analysis
Source: Lancet Public Health. 2021 Feb 10;6(3):e164–74. doi: 10.1016/S2468-2667(20)30233-4 (PMC7907684; doi:10.1016/S2468-2667(20)30233-4)
Supplement: Supplementary appendix [file mmc1.pdf]

THE LANCET
Public Health

Supplementary appendix

This appendix formed part of the original submission and has been peer reviewed. We post it as supplied by the authors.

Supplement to: Zhong S, Senior M, Yu R, et al. Risk factors for suicide in prisons: a systematic review and meta-analysis. *Lancet Public Health* 2021; published online Feb 9. http://dx.doi.org/10.1016/S2468-2667(20)30233-4.

Appendix. Search databases and results.

**Databases**	**Records**
GLOBAL HEALTH (OVID)	**299**
MEDLINE (OVID)	**1584**
PSYCINFO (OVID)	**2198**
EMBASE (OVID)	**3031**
CINAHL (EBSCO)	**921**
Other resources	**8**
**TOTAL**	**8041**

Appendix. Full search strategy for each database

GLOBAL HEALTH

1 exp Suicide/

2 suicid*.mp. [mp=title, abstract, original title, name of substance word, subject heading word, floating sub-heading word, keyword heading word, organism supplementary concept word, protocol supplementary concept word, rare disease supplementary concept word, unique identifier, synonyms]

3 suicid*.ab. or suicid*.ti.

4 #1 or #2 or #3

5 (prison* or felon* or detain* or jail* or custod* or her majesty's prison or HMP or remand* or offender* or institution or penal or inmate* or correction* or sentenced or incarcerat* or gaol*).ab. or (prison* or felon* or detain* or jail* or custod* or her majesty's prison or HMP or remand* or offender* or institution or penal or inmate* or correction* or sentenced or incarcerat* or gaol*).ti.

6 (prison* or felon* or detain* or jail* or custod* or her majesty's prison or HMP or remand* or offender* or institution or penal or inmate* or correction* or sentenced or incarcerat* or gaol*).mp. [mp=title, abstract, original title, name of substance word, subject heading word, floating sub-heading word, keyword heading word, organism supplementary concept word, protocol supplementary concept word, rare disease supplementary concept word, unique identifier, synonyms]

7 exp Prisons/

8 #5 or #6 or #7

9 #4 and #8

#10 limit #9 to yr="inception - Current"

MEDLINE

1 exp Suicide/

2 suicid*.mp. [mp=title, abstract, original title, name of substance word, subject heading word, floating sub-heading word, keyword heading word, organism supplementary concept word, protocol supplementary concept word, rare disease supplementary concept word, unique identifier, synonyms]

3 suicid*.ab. or suicid*.ti.

4 #1 or #2 or #3

5 (prison* or felon* or detain* or jail* or custod* or her majesty's prison or HMP or remand* or offender* or institution or penal or inmate* or correction* or sentenced or incarcerat* or gaol*).ab. or (prison* or felon* or detain* or jail* or custod* or her majesty's prison or HMP or remand* or offender* or institution or penal or inmate* or correction* or sentenced or incarcerat* or gaol*).ti.

6 (prison* or felon* or detain* or jail* or custod* or her majesty's prison or HMP or remand* or offender* or institution or penal or inmate* or correction* or sentenced or incarcerat* or gaol*).mp. [mp=title, abstract, original title, name of substance word, subject heading word, floating sub-heading word, keyword heading word, organism supplementary concept word, protocol supplementary concept word, rare disease supplementary concept word, unique identifier, synonyms]

7 exp Prisons/

8 #5 or #6 or #7

9 #4 and #8

10 limit #9 to yr="2006 -Current"

PSYCHINFO

1 exp Suicide/

2 suicid*.mp. [mp=title, abstract, original title, name of substance word, subject heading word, floating sub-heading word, keyword heading word, organism supplementary concept word, protocol supplementary concept word, rare disease supplementary concept word, unique identifier, synonyms]

3 suicid*.ab. or suicid*.ti.

4 #1 or #2 or #3

5 (prison* or felon* or detain* or jail* or custod* or her majesty's prison or HMP or remand* or offender* or institution or penal or inmate* or correction* or sentenced or incarcerat* or gaol*).ab. or (prison* or felon* or detain* or jail* or custod* or her majesty's prison or HMP or remand* or offender* or institution or penal or inmate* or correction* or sentenced or incarcerat* or gaol*).ti.

6 (prison* or felon* or detain* or jail* or custod* or her majesty's prison or HMP or remand* or offender* or institution or penal or inmate* or correction* or sentenced or incarcerat* or gaol*).mp. [mp=title, abstract, original title, name of substance word, subject heading word, floating sub-heading word, keyword heading word, organism supplementary concept word, protocol supplementary concept word, rare disease supplementary concept word, unique identifier, synonyms]

7 exp Prisons/

8 #5 or #6 or #7

9 #4 and #8

10 limit #9 to yr="2006 -Current"

EMBASE

1 exp Suicide/

2 suicid*.mp. [mp=title, abstract, original title, name of substance word, subject heading word, floating sub-heading word, keyword heading word, organism supplementary concept word, protocol supplementary concept word, rare disease supplementary concept word, unique identifier, synonyms]

3 suicid*.ab. or suicid*.ti.

4 #1 or #2 or #3

5 (prison* or felon* or detain* or jail* or custod* or her majesty's prison or HMP or remand* or offender* or institution or penal or inmate* or correction* or sentenced or incarcerat* or gaol*).ab. or (prison* or felon* or detain* or jail* or custod* or her majesty's prison or HMP or remand* or offender* or institution or penal or inmate* or correction* or sentenced or incarcerat* or gaol*).ti.

6 (prison* or felon* or detain* or jail* or custod* or her majesty's prison or HMP or remand* or offender* or institution or penal or inmate* or correction* or sentenced or incarcerat* or gaol*).mp. [mp=title, abstract, original title, name of substance word, subject heading word, floating sub-heading word, keyword heading word, organism supplementary concept word, protocol supplementary concept word, rare disease supplementary concept word, unique identifier, synonyms]

7 exp Prisons/

8 #5 or #6 or #7

9 #4 and #8

10 limit #9 to yr="2006 -Current"

CINAHL

1 exp Suicide/

2 suicid*.su.

3 suicid*.ab. or suicid*.ti.

4 #1 or #2 or #3

5 (prison* or felon* or detain* or jail* or custod* or her majesty's prison or HMP or remand* or offender* or institution or penal or inmate* or correction* or sentenced or incarcerat* or gaol*).ab. or (prison* or felon* or detain* or jail* or custod* or her majesty's prison or HMP or remand* or offender* or institution or penal or inmate* or correction* or sentenced or incarcerat* or gaol*).ti.

6 (prison* or felon* or detain* or jail* or custod* or her majesty's prison or HMP or remand* or offender* or institution or penal or inmate* or correction* or sentenced or incarcerat* or gaol*).mp. [mp=title, abstract, original title, name of substance word, subject heading word, floating sub-heading word, keyword heading word, organism supplementary concept word, protocol supplementary concept word, rare disease supplementary concept word, unique identifier, synonyms]

7 exp Prisons/

8 #5 or #6 or #7

9 #4 and #8

10 limit #9 to yr="2006 -Current"

Appendix Figure 1. Suicide risk in people in prison and demographic and institutional factors

A. Demographic factors

Risk factor	Number of Studies	Cases, n/N	Controls, n/N	Pooled OR (95%CI)	z	p–value	I2(%)
Homeless	4	55/318	104/851	2.4 (0.3–19.8)	0.8	.43	93.5
White	22	8117/11773	5766/12112	2.0 (1.4–2.7)	4.2	<.0001	94.7
Married	10	518/1800	500/2263	1.5 (1.2–1.7)	5.2	<.0001	0
Employed	5	317/673	521/1224	1.3 (0.5–3.2)	0.5	.59	94.7
Male gender	37	18819/20080	18616/20263	1.2 (1.0–1.5)	2.1	.036	59.2
Age 50+	10	635/4736	535/4736	1.2 (1.0–1.4)	2.4	.017	22.5
Age 31–40	10	767/2448	716/2448	1.1 (1.0–1.2)	1.5	.12	7.8
Age 41–50	9	425/2370	389/2370	1.1 (0.9–1.3)	0.5	.59	36.3
Education, not continued after age 16	3	235/449	430/850	0.9 (0.4–2.4)	–0.2	.87	91.1
Age 21–30	6	402/1075	423/1075	0.9 (0.8–1.2)	–0.6	.56	19.1
Age16–20	5	91/617	103/617	0.8 (0.6–1.2)	–1.0	.32	8.0
Age 18–29	5	726/2279	868/2279	0.7 (0.7–0.8)	–4.6	<.0001	0
Hispanic	6	1200/8694	1996/8694	0.6 (0.5–0.8)	–3.8	<.0001	68.1
Black	17	1786/11895	4551/11901	0.4 (0.3–0.5)	–7.8	<.0001	86.9

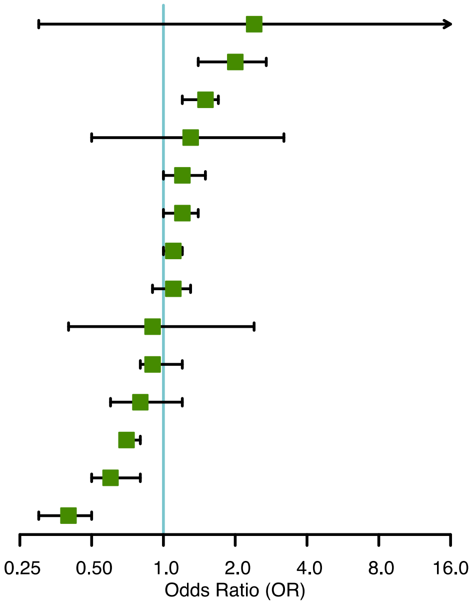

B. Institutional factors

Risk factor	Number of Studies	Cases, n/N	Controls, n/N	Pooled OR (95%CI)	z	p–value	I2(%)
Single cell occupancy	3	310/435	167/519	6.8 (2.3–19.8)	3.5	.0004	89.6
No social visits	3	445/750	426/907	1.9 (1.5–2.4)	4.3	<.0001	19.5

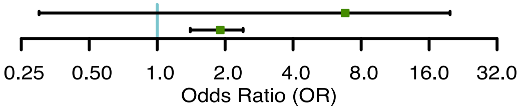

Note: n = number with risk factor. N = total number of cases/controls.

Appendix Table 1. Characteristics of updated studies on risk factors associated with suicide in prison

First author, year	Location	Period	Population studied	Control group	Cases, N	Age (yrs)
Group 1 study (case-control)						
Humber (2013)[1]	England and Wales	2005-2008	A consecutive case series of suicides in all prisons in England and Wales	Matched for gender, age, establishment type and date of reception into prison.	220	15-67
Group 2 studies (compare to average or total prison population)						
Austin (2014)[2]	Australia	1996-2010	All suicides in South Australian prisons	TPP (mid year) External control	21	17-80
Baillargeon (2009)[3]	United States	2006-2007	All suicides within the Texas Department of Criminal Justice	TPP	41	≥16
Bedoya (2009)[4]	Spain	1990-2005	All suicides in prisons in Catalonia (included those occurring during leave/in hospital)	AAP	65	Not reported
Betz (2011)[5]	United States	2004-2008	All suicides in Colorado prisons	TPP	64	Not reported
Bird (2008)[6]	Scotland	1994-2003	All suicides in male prisons in Scotland	ADP External control	108	≥15
Brittain (2013)/NY correctional services[7]	United States	2001-2009	All suicides in NY state facilities	TPP (mid-year)	121	≥16
Carson (2020)a[8]	United States	2000-2016	All suicides in local jails in USA	ADP	5207	Youngest≤17, oldest≥55
Carson (2020)b[9]	United States	2001-2016	All suicides in state prisons in USA	Person-years in prison	3300	Youngest≤17, oldest≥55

Castelpietra (2018)[10]	Italy	2010-2016	All male suicide cases in North-East Italian prisons	AAP	29	≥18
Choi (2019)[11]	United States	2005-2014	All suicides in prisons in the USA	TPP<br>External control	1727	14-82
Daniel (2006)[12]	United States	1992-2002	All suicides in prisons in the USA	TPP<br>External control	37	18-53
Duthe (2013)[13]	France	2006-2009	All suicides in male prisons in France	Person-years	353	≥18
Duthe (2014)[14]	France	2006-2009	All suicides in French prisons	Person-years	377	Across all age
Esposito (2017)[15]	Italy	2002-2015	All suicides in Italian prisons	ADP	716	18-72
Favril (2018)[16]	Belgium	2000-2016	All suicides in Belgian prisons	AAP<br>External control	262	19-72
Fazel (2017)[17]	24 countries in Europe, Australia, and North America	2011-2014	All suicides from any prison	AAP	2810	Not reported
Fritz (2020)[18]	10 countries in South America	2000-2017	Suicides in all prisons in Brazil, Chile, Colombia, Ecuador, Guyana, Paraguay, Peru, Uruguay; Federal prisons in Argentina; a single maximum security prison in Bolivia	Person-years in prison	1324	Not reported
Gauthier (2015)[19]	Switzerland	2000-2010	All suicides in Swiss prisons	TPP (mid-year)<br>External control	50	19-60
Hawton (2014)[20]	England and Wales	2004-2009	All suicides in prisons in England and Wales	TPP	455	≥15
Hayes (2012)[18]	United States	2005-2006	Suicides in 205 detention facilities and 52 holding facilities in US	TPP<br>External control	464	Youngest <17
Huey (2008)[22]	United States	2000	All suicides in state and private facilities (excludes federal)	ADP	172	Not reported
Humber (2011)[23]	England and Wales	1999-2007	All suicides in prisons in England and Wales	AAP	766	15-75

Kim (2007)[24]	United States	1995-2004	Suicides in all prisons in USA	ADP	19	Not reported
Morthorst (2020)[25]	Denmark, Norway, Iceland	2000-2016	All suicides in prisons in Denmark, Norway and Iceland	Person-years in prison	167	15 - >55
Mumola (2005)[26]	United States	2000-2002	Suicides in all prisons in USA	ADP	1770	Youngest<18, oldest ≥55
O'Driscroll (2007)[27]	Australia	1995-2005	All suicides in New South Wales prisons	ADP (mid-year) External control	92	≥18
Opitz-Welke (2013)[28]	Germany	2000-2011	All suicides in German prisons	TPP	960	Youngest<20, oldest>60
Opitz-Welke (2016)[29]	Germany	2000-2013	All suicides in German prisons (pretrial and criminal custody)	TPP	1067	Not reported
Opitz-Welke (2019)[30]	Germany	2000-2013	All suicides in German prisons	TPP	1067	Not reported
Patterson (2008)[31]	United States	1999-2004	Suicides in all prisons in USA	TPP (mid-year) External control	154	Youngest<18, oldest>50
Preti (2006)[32]	Italy	1990-2002	All suicides in Italian prisons	ADP	642	18 – 65
Radeloff (2015)[33]	Germany	2000-2010	All suicides in German prisons	Person-years	860	≥14
Radeloff (2017)[34]	Germany	2000-2013	All suicides of male sentenced prisoners (not including pre-trial)	Person-years	425	14-59
Radeloff (2019)[35]	Germany	2000 - 2016	All suicides of male sentenced prisoners (not including pre-trial)	Person-years	524	≥14
Reeves (2014)[36]	United States	2005-2011	All suicides in the New Jersey Department of Corrections	Total beds	26	Not reported
Rivlin (2012)[37]	England and Wales	2007-2009/1995-2009	All suicide in prisons in England and Wales	TPP (mid-year) External control	121	≥18
Rosen (2011)[38]	United States	1995-2005	All prisoners in North Carolina state prison	TPP	31	20-79
Safer Custody (2015)[39]	England and Wales	1978-2014	All suicides in prisons in England and Wales	AAP	706	≥15
Safer Custody (2020)[40]	England and Wales	2015-2019	All suicides in prisons in England and Wales	AAP	463	≥15

Thomas (2018)[41]	United States	2000-2014	All suicides in local jails	TPP<br>External control	4508	Youngest<17, oldest>55
Voulgaris (2019)[42]	Germany	2012-2017	All suicides in prisons in Berlin	Total prison population<br>External control	24	Not reported
Wobeser (2002)[43]	United States	1990-1999	All suicides in federal penitentiaries and provincial prisons, and police cells in Ontario	ADP<br>External control	101	Not reported

Abbreviations: AAP = average annual prison population (population at a predefined date in the year), ADP = average daily prison population (mean prison population, such as July 1 in the year), JIP = jail inmate profile, TPP = total prison population (total population at specific timepoint, for example, time of study. Unless otherwise stated, population consisted of remand/pretrial and sentenced prisoners.

Appendix Table 2 Included studies from original review

First author, year	Location	Period	Population studied	Control group	Cases, N	Controls, N
Group 1 studies						
Blaauw (2005)[44]	The Netherlands	1987–1998	Suicides in detained and sentenced prisoners in the Netherlands (including jails, prisons and 10 treatment institutions)	Randomly selected jail inmates	95	247
Bourgoin (1993)[45]	France	1990-1992	Suicides in prisons (pretrial and sentenced)	Randomly selected inmates	179	360
Dahle (2005)[46]	Germany	1991-2000	Suicides of pretrial detainees in a Berlin prison	Matched by date of reception same prison	30	30
Fruehwald (2004)[47]	Austria	1975-1999	Suicides of pretrial, sentenced or mentally disordered prisoners in correctional institutions.	Matched for age, gender, nationality, institution and sentence	220	440
Kerkhof (1990)[48]	The Netherlands	1973-1984	Suicides of detained or sentenced prisoners	Randomly selected controls	44	54
Lupei (1981)[49]	USA	1977-1979	Suicides in Oklahoma state jails	For each case, control = last prisoner released on prior day	21	21
Phillips (1986)[50]	UK	1973-1983	Suicides of male prisoners in a London prison	One in 200 index cards from prison population 1975 onwards	34	214
Winter (2003)[51]	USA	1980-1998	Suicides of detained or sentenced prisoners in county jail	Random sample jail admissions	103	104
Group 2 studies (total or average prison population control)						
Anno (1985)[52]	USA	1980-1985	All suicides in Texas Department of Corrections prisons	ADP	38	

Backett (1987)[53]	Scotland	1970-1982	Suicides of remand and sentenced prisoners in Scotland	TPP	33	
Bogue (1995)[54]	Scotland	1976-1993	All suicides in Scottish prisons	ADP 1976-1979, 1980-1983	83	
Crighton (1997)[55]	England, Wales	1988-1990 and 1994-1995	All suicides in prisons in England and Wales	ADP	197	
Scott-Denoon (1984)[56]	Canada	1970-1980	All suicides in British Columbia correctional facilities (remand and sentenced)	TPP	35	
Dooley (1990)[57]	England, Wales	1972-1987	All suicides in prisons in England and Wales (including remand and youth custody centers)	AAP	295	
DuRand (1995)[58]	USA	1967-1992	All suicides in a large (1700-bed) jail in Detroit	TPP	37	
Frickey (1999)[59]	USA	1993-1997	All suicides in federal prisons	TPP 1997	61	
Fruehwald (2000)[60]	Austria	1975-1997	All suicides in correctional facilities	ADP	220	
Hatty (1986)[61]	Australia	1980-1985	All suicides in Australian prisons	TPP	77	
Hayes (1989)[62]	USA	1979-1986	All suicides in county jails and city/police department lockups	Jail inmate profile 1983	854	
Hurley (1989)[63]	Australia	1973-1987	All suicides in prisons in Brisbane	AAP	20	
Kovasznay (2004)[64]	USA	1993-1999	All suicides in New York State prisons	TPP	76	
Laishes (1997)[65]	Canada	1992-1996	All suicides in federal prisons	TPP 1994	66	
New York State Medical Review Board (1998)[66]	USA	1993-1997	All suicides in in state correctional facilities in New York	TPP	50	
National Association for the	England, Wales	1980-1989	All suicides in prisons in England and Wales	ADP	242	

Care and Resettlement of Offenders (1990)[67]						
Safer Custody Group (2005)[68]	England, Wales	2004-2005	All suicides in prisons in England and Wales	TPP	85	
Safer Custody Group (2007)[69]	England, Wales	1999-2004	All suicides in prisons in England and Wales	TPP	442	
Salive (1989)[70]	USA	1979-1987	All suicides in Maryland prisons, Baltimore – suicides in prisoners convicted and sentenced to ≥1 year of prison	TPP census	37	
Snow (2002)[71]	England, Wales	1996-2001	All suicides in prisons in England and Wales	TPP	451	
Tatarelli (1999)[72]	Italy	1996-1997	All suicides in prisons in Italy	TPP 1996-1997	100	
Towl (1998)[73]	England, Wales	1988 - 1995	All suicides in prisons in England and Wales	TPP	377	
White (2002)[74]	USA	1993-1997	All suicides in federal prisons	TPP	62	
He (2001)[75]	USA	1996-1997	All suicides in 20 of 107 institutional division prison units of Texas criminal justice system	TPP	25	
Skegg (1993)[76]	New Zealand	1973 - 1988	Suicides in all prisons in New Zealand (remand and custodial)	TPP receptions[a]	39	
Novick (1978)[77]	USA	1971-1976	Suicides in in New York City correctional facilities	1420 admissions in 1975	52	

Notes: [a] receptions = number of prisoners entering prison. Abreviations: AAP = average annual prison population (at a defined date in the year, such as 1 July in the year), ADP = average daily prison population/mean prison population, TPP = total prison population (total population at specific timepoint, for example, time of study).

Appendix Table 3. Characteristics of updated studies on additional risk factors associated with suicide in prison

Categories	Risk factor	Study group [a]	Number of Studies	n, case	N, case	n, control	N, control	Odds Ratio	95% CI	z	p-value	I^2(%)
Demographic	Age 25+[1]	2	8	12019	14714	11648	14714	1.2	0.9-1.7	1.19	.24	95.1
	Age 30+[3,9,12]	2	3	1344	2142	1352	2142	1.2	0.8-1.9	0.74	.46	81.0
	Age 40+[28]	2	1	353	960	288	960	1.4	1.2-1.7	3.61	<.0003	-
	Age 45+[6,7,27,41]	2	6	3003	13324	2679	13324	0.8	0.6-1.1	-1.29	.20	95.9
	Catholic religion[47]	1	1	165	219	319	404	0.8	0.6-1.2	-1.03	.30	-
	Not being a citizen of the country of incarceration[4,10,14–16,19,27,30,32,40]	2	10	885	4133	998	4133	0.7	0.6-1.0	-2.27	.023	73.7
Criminal history	Length of sentence less than 6 mo[27]	2	1	29	40	37	40	0.2	0.1-0.8	-2.21	.027	-
	Length of sentence less than 12 mo[14,39,40]	2	3	185	1024	154	1024	1.3	0.8-2.0	1.19	.23	69.4
	Length of sentence less than 48 mo[13]	2	1	115	205	125	205	0.8	0.6-1.2	-1.00	.32	-
	Longer than 1 yr served[7,48]	2	2	49	165	50	175	2.2	0.1-67.1	0.44	.66	97.0
	Previous violent conviction[1]	1	1	92	209	48	218	2.8	1.8-4.2	4.77	<.0001	-
Clinical	History of drug misuse[1]	1	1	101	220	100	220	1.0	0.7-1.5	0.10	.92	-
	History of psychiatric diagnosis[1]	1	1	101	206	42	220	4.1	2.6-6.3	6.36	<.0001	-
	Referred to psychiatrist during	1	1	95	220	30	214	4.7	2.9-7.5	6.43	<.0001	-

	prison term[11]											
	Previous psychiatric inpatient admission[1]	1	1	54	208	14	219	5.1	2.8-9.6	5.14	<.0001	-
	Hospitalization[14]	2	1	131	377	86	377	1.8	1.3-2.5	3.60	.0003	-
	Criminally not responsible[16]	2	1	47	153	12	153	5.2	2.6-10.3	4.74	<.001	-
	Mental health concerns documented at reception[1]	1	1	65	220	11	220	8.0	4.1-15.6	6.05	<.0001	-
	Self-harm risk document open[1]	1	1	118	220	31	220	7.1	4.4-11.2	8.27	<.0001	-
	Previous MHS contact[1]	1	1	104	220	39	220	4.2	2.7-6.4	6.41	<.0001	-
**Institutional**	High secure cell[10]	2	1	1	29	3	29	1.0	0.1-16.8	0.00	1.00	-
	Adjudication[1]	1	1	74	211	64	220	1.3	0.9-2.0	1.33	.18	-
	Disciplinary cell[14]	2	1	30	377	3	377	10.8	3.3-35.6	3.90	<.0001	-

[a] group 1 studies using a randomly selected or matched control group and group 2 studies using the total/average prison population during a matched period.

Appendix Table 4. OHAT scale for assessment of quality of included studies.

Studies	Comparison Group	Confounding*	Complete outcome data	Exposure characterization	Outcome assessment	Outcome reporting	Other threats
Anno (1985)	+	-	++	+	+	++	-
Austin (2014)	+	-	++	+	+	++	-
Backett (1987)	+	-	+	-	-	+	+
Baillargeon (2009)	++	+	+	++	+	++	+
Bedoya (2009)	+	-	+	+	+	++	-
Betz (2011)	+	-	+	+	+	++	+
Bird (2008)	+	-	++	+	+	++	+
Blaauw (2005)	++	-	++	+	+	++	++
Bogue (1995)	+	+	+	++	++	+	+
Bourgoin (1993)	+	+	+	++	++	+	+
Brittain (2013)/NY correctional services	+	-	-	+	++	++	+
Carson (2020)	+	-	+	+	+	++	+
Carson (2020)	+	-	+	+	+	++	+
Castelpietra (2018)	+	-	+	+	-	+	+
Choi (2019)	+	-	+	+	++	++	+
Crighton (1997)	+	-	-	-	-	-	-
Dahle (2005)	++	+	+	+	+	+	+
Daniel (2006)	+	-	+	+	+	++	-
Dooley (1990)	+	+	++	++	++	++	+
DuRand (1995)	+	+	-	-	+	+	-
Duthe (2013)	++	++	-	+	+	++	+
Duthe (2014)	++	++	++	+	+	++	+
Esposito (2017)	-	-	+	+	+	+	-
Favril (2018)	+	-	++	+	++	++	+
Fazel (2017)	+	+	+	+	-	++	+
Frickey (1999)	+	+	+	+	+	++	+
Fritz (2020)	+	-	-	+	-	+	+
Fruehwald (2000)	+	+	+	+	+	++	+
Fruehwald (2004)	++	++	++	+	++	++	++
Gauthier (2015)	+	-	++	+	++	++	+
Hatty (1986)	+	+	+	+	++	++	+
Hawton (2014)	+	+	+	+	++	++	+
Hayes (1989)	+	+	+	+	+	++	+
Hayes (2012)	+	-	++	+	++	++	-
He (2001)	+	+	++	+	++	++	-
Huey (2008)	+	-	+	+	-	++	+
Humber (2011)	+	+	+	+	++	++	+
Humber (2013)	++	++	++	+	++	++	++
Hurley (1989)	+	-	+	-	+	+	+

Kerkhof (1990)	++	+	++	+	+	++	++
Kim (2007)	+	-	++	+	++	++	-
Kovasznay (2004)	+	+	+	+	+	+	+
Laishes (1997)	+	-	-	-	-	-	-
Lupei (1981)	++	+	++	++	+	++	+
Morthorst (2020)	+	-	+	+	-	++	+
Mumola (2005)	+	-	+	+	+	++	+
National Association for the Care and Resettlement of Offenders (1990)	+	-	+	+	+	++	+
New York State Medical Review Board (1998)	+	-	+	+	+	++	+
Novick (1978)	+	+	++	+	++	++	+
O'Driscroll (2007)	+	-	++	+	++	++	+
Opitz-Welke (2013)	+	-	+	+	+	++	+
Opitz-Welke (2016)	+	-	+	+	+	++	-
Opitz-Welke (2019)	+	-	+	+	+	++	+
Patterson (2008)	+	-	+	+	+	++	+
Phillips (1986)	+	-	+	+	+	++	+
Preti (2006)	+	-	+	+	++	++	+
Radeloff (2015)	+	+	-	+	+	++	+
Radeloff (2017)	+	+	+	+	+	++	+
Radeloff (2019)	+	+	+	+	+	++	+
Reeves (2014)	-	-	-	+	+	++	+
Rivlin (2012)	+	-	+	+	++	++	+
Rosen (2011)	+	+	+	+	++	++	+
Safer Custody (2015)	+	-	+	+	++	++	+
Safer Custody (2020)	+	-	+	+	++	++	+
Salive (1989)	+	+	++	++	++	++	+
Scott-Denoon (1984)	+	-	+	+	++	++	+
Skegg (1993)	+	-	+	+	++	++	+
Snow (2002)	+	-	+	+	++	++	+
Tatarelli (1999)	+	-	-	-	-	-	-
Thomas (2018)	+	-	+	+	+	++	+
Towl (1998)	+	+	++	++	+	++	+
Voulgaris (2019)	+	-	+	+	++	++	+
White (2002)	+	+	++	+	+	++	+
Winter (2003)	+	+	++	+	+	+	+
Wobeser (2002)	+	-	+	+	++	++	-

Note: ++ refers to Definitely Low, + refers to Probably Low, - refers to Probably High, - - refers to Definitely High. *A definitely/probably low risk of bias for confounding indicates there was adjustment for in some part of the study design or analysis, it does not indicate that adjusted odds ratios were reported, or could be extracted from the studies.

Appendix Table 5. Differences between adjusted and crude odds ratio on identified risk factors.

Risk factor	Number of Studies	Adjusted odds ratio (95% CI)	Crude odds ratio (95% CI)	Q	p-value
Married	10	1.4 (1.0-1.9)	1.5 (1.2-1.8)	0.1	.751
Employed	5	0.5 (0.3-0.8)	2.7 (1.7-4.4)	25.1	<.0001
Previous conviction	5	0.9 (0.7-1.3)	2.3 (0.6-8.6)	1.7	.190
Current psychiatric diagnosis	7	5.3 (3.2-8.8)	7.1 (2.6-19.1)	0.3	.604
History of self-harm	5	6.3 (4.0-10.2)	9.1 (2.8-29.8)	0.3	.572
Single cell occupancy	3	4.7 (1.5-14.5)	30.4 (5.2-178.4)	3.0	.0817
No social visits	3	1.6 (1.2-2.1)	2.3 (1.6-3.1)	2.5	.115

References:

1 Humber N, Webb R, Piper M, Appleby L, Shaw J. A national case–control study of risk factors among prisoners in England and Wales. *Soc Psychiatry Psychiatr Epidemiol* 2013; **48**: 1177–85.

2 Austin AE, van den Heuvel C, Byard RW. Prison Suicides in South Australia: 1996-2010. *J Forensic Sci* 2014; **59**: 1260–2.

3 Baillargeon J, Penn JV, Thomas CR, Temple JR, Baillargeon G, Murray OJ. Psychiatric Disorders and Suicide in the Nation's Largest State Prison System. *J Am Acad Psychiatry Law* 2009; **37**: 6.

4 Bedoya A, Martínez-Carpio PA, Humet V, Leal MJ, Lleopart N. Incidencia del suicidio en las prisiones de Cataluña: análisis descriptivo y comparado. *Rev Esp Sanid Penit* 2009; **11**. DOI:10.4321/S1575-06202009000200002.

5 Betz ME, Krzyzaniak SM, Hedegaard H, Lowenstein SR. Completed Suicides in Colorado: Differences between Hispanics and Non-Hispanic Whites: Hispanic Ethnicity and Suicide in Colorado. *Suicide Life Threat Behav* 2011; **41**: 445–52.

6 Bird SM. Changes in male suicides in Scottish prisons: 10-year study. *Br J Psychiatry* 2008; **192**: 446–9.

7 Brittain J, Axelrod G, Venters H. Deaths in New York City Jails, 2001–2009. *Am J Public Health* 2013; **103**: 638–40.

8 Carson EA, Cowhig MP. Mortality in Local Jails, 2000-2016 - Statistical Tables. *Stat Tables* 2020; : 30.

9 Carson EA, Cowhig MP. Mortality in State and Federal Prisons, 2001-2016 - Statistical Tables. *Stat Tables* 2020; : 25.

10 Castelpietra G, Egidi L, Caneva M, *et al.* Suicide and suicides attempts in Italian prison epidemiological findings from the "Triveneto" area, 2010–2016. *Int J Law Psychiatry* 2018; **61**: 6–12.

11 Choi NG, DiNitto DM, Marti CN. Suicide Decedents in Correctional Settings: Mental Health Treatment for Suicidal Ideation, Plans, and/or Attempts. *J Correct Health Care* 2019; **25**: 70–83.

12 Daniel AE, Fleming J. Suicides in a State Correctional System, 1992-2002: A Review. *J Correct Health Care* 2006. DOI:10.1177/1078345806287541.

13 Duthe G, Hazard A, Kensey A, Shon JL. Suicide among male prisoners in France: a prospective population-based study. *Forensic Sci Int* 2013; **233**: 273–7.

14 Duthé G, Hazard A, Kensey A, Wiles-Portier E. Trends and risk factors for prisoner suicide in France. *Population* 2014; **69**: 463–493.

15 Esposito M. Suicidal Risk in Italian Prisons. A Population-Based Cohort Study. *Sociol Mind* 2018; **8**: 46–69.

16 Favril L, Wittouck C, Audenaert K, Vander Laenen F. A 17-Year National Study of Prison Suicides in Belgium. *Crisis* 2019; **40**: 42–53.

17 Fazel S, Ramesh T, Hawton K. Suicide in prisons: an international study of prevalence and contributory factors. *Lancet Psychiatry* 2017; **4**: 946–52.

18 Fritz FD, Fazel S, Benavides Salcedo A, *et al.* 1324 prison suicides in 10 countries in South America: incidence, relative risks, and ecological factors. *Soc Psychiatry Psychiatr Epidemiol* 2020; published online May 13. DOI:10.1007/s00127-020-01871-3.

19 Gauthier S, Reisch T, Bartsch C. Swiss Prison Suicides Between 2000 and 2010: Can We Develop New Prevention Strategies Based on Detailed Knowledge of Suicide Methods? *Crisis* 2015; **36**: 110–6.

20 Hawton K, Linsell L, Adeniji T, Sariaslan A, Fazel S. Self-harm in prisons in England and Wales: an epidemiological study of prevalence, risk factors, clustering, and subsequent suicide. *Lancet* 2014; **383**: 1147–54.

21 Hayes LM. National Study of Jail Suicide: 20 Years Later. *J Correct Health Care* 2012; **18**: 233–45.

22 Huey MP. Deprivation, importation, and prison suicide. 2008; published online Aug. http://athenaeum.libs.uga.edu/handle/10724/24926 (accessed Feb 23, 2020).

23 Humber N, Piper M, Appleby L, Shaw J. Characteristics of and trends in subgroups of prisoner suicides in England and Wales. *Psychol Med* 2011; **41**: 2275–85.

24 Kim S, Ting A, Puisis M, *et al.* Deaths in the Cook County Jail: 10-Year Report, 1995–2004. *J Urban Health* 2007; **84**: 70–84.

25 Morthorst BR, Mehlum L, Pàlsson SP, *et al.* Suicide Rates in Nordic Prisons 2000–2016. *Arch Suicide Res* 2020; : 1–11.

26 Mumola CJ. Suicide And Homicide In State Prisons And Local Jails. 2005. DOI:10.1037/e479702006-001.

27 O'Driscoll C, Samuels A, Zacka M. Suicide in New South Wales Prisons, 1995–2005: Towards a Better Understanding. *Aust N Z J Psychiatry* 2007; **41**: 519–24.

28 Opitz-Welke A, Bennefeld-Kersten K, Konrad N, Welke J. Prison suicides in Germany from 2000 to 2011. *Int J Law Psychiatry* 2013; **36**: 386–9.

29 Opitz-Welke A, Bennefeldt-Kersten K, Konrad N, Welke J. Prison suicide in female detainees in Germany 2000–2013. *J Forensic Leg Med* 2016; **44**: 68–71.

30 Opitz-Welke A, Konrad N, Welke J, Bennefeld-Kersten K, Gauger U, Voulgaris A. Suicide in Older Prisoners in Germany. *Front Psychiatry* 2019; **10**: 154.

31 Patterson RF, Hughes K. Review of Completed Suicides in the California Department of Corrections and Rehabilitation, 1999 to 2004. *Psychiatr Serv* 2008; **59**: 676–682.

32 Preti A, Cascio MT. Prison Suicides and Self-harming Behaviours in Italy, 1990-2002. *Med Sci Law* 2006; **46**: 127–34.

33 Radeloff D, Lempp T, Herrmann E, Kettner M, Bennefeld-Kersten K, Freitag CM. National total Survey of German adolescent Suicide in Prison. *Eur Child Adolesc Psychiatry* 2015; **24**: 219–25.

34 Radeloff D, Lempp T, Kettner M, Rauf A, Bennefeld-Kersten K, Freitag CM. Male suicide rates in German prisons and the role of citizenship. *PLOS ONE* 2017; **12**: e0178959.

35 Radeloff D, Stoeber F, Lempp T, Kettner M, Bennefeld-Kersten K. Murderers or thieves at risk? Offence-related suicide rates in adolescent and adult prison populations. *PLOS ONE* 2019; **14**: e0214936.

36 Reeves R, Tamburello A. Single Cells, Segregated Housing, and Suicide in the New Jersey Department of Corrections. *J Am Acad Psychiatry Law* 2014; **42**: 5.

37 Rivlin A, Fazel S, Marzano L, Hawton K. Studying survivors of near-lethal suicide attempts as a proxy for completed suicide in prisons. *Forensic Sci Int* 2012; **220**: 19–26.

38 Rosen DL, Wohl DA, Schoenbach VJ. All-Cause and Cause-Specific Mortality Among Black and White North Carolina State Prisoners, 1995–2005. *Ann Epidemiol* 2011; **21**: 719–26.

39 Safer Custody Group. Analysis of Self-inflicted deaths in custody in England and Wales between 1978 and March 2014. London, United Kingdom: National Offender Management Service, 2015.

40 Safer Custody Group. Deaths in custody in England Wales, 1978-2019. London, United Kingdom: National Statistics, 2020 https://www.gov.uk/government/collections/safety-in-custody-statistics (accessed Feb 23, 2020).

41 Thomas AL, Scott J, Mellow J. The validity of open-source data when assessing jail suicides. *Health Justice* 2018; **6**: 11.

42 Voulgaris A, Hartwig S, Konrad N, Opitz-Welke A. Influence of drugs on prison suicide - A retrospective case study. *Int J Law Psychiatry* 2019; **66**: 101460.

43 Wobeser WL, Datema J, Bechard B, Ford P. Causes of death among people in custody in Ontario, 1990–1999. 2002; **167**: 1109–13.

44 Blaauw E, Kerkhof AJ, Hayes LM. Demographic, criminal, and psychiatric factors related to inmate suicide. *Suicide Life Threat Behav* 2005; **35**: 63–75.

45 Bourgoin N. La mortalité par suicide en prison. *Rev Epidem Santé Publ* 1993; **41**: 146–54.

46 Dahle K-P, Lohner JC, Konrad N. Suicide prevention in penal institutions: Validation and optimization of a screening tool for early identification of high-risk inmates in pretrial detention. *Int J Forensic Ment Health* 2005; **4**: 53–62.

47 Fruehwald S, Matschnig T, Koenig F, Bauer P, Frottier P. Suicide in custody: case–control study. *Br J Psychiatry* 2004; **185**: 494–498.

48 Kerkhof AJ, Bernasco W. Suicidal behavior in jails and prisons in the Netherlands: Incidence, characteristics, and prevention. *Suicide Life Threat Behav* 1990; **20**: 123–137.

49 Lupei RA. Jail suicides: Demographic and behavioral factors postdictive of the completed act. Oklahoma State University, 1981.

50 Phillips M. A study of suicides and attempted suicides at HMP Brixton, 1973-1983. Home Office: Home Office, 1986.

51 Winter MM. County jail suicides in a Midwestern state: Moving beyond the use of profiles. *Prison J* 2003; **83**: 130–148.

52 Anno BJ. Patterns of suicide in the Texas Department of Corrections 1980–1985. *J Prison Jail Health* 1985.

53 Backett SA. Suicide in Scottish prisons. *Br J Psychiatry* 1987; **151**: 218–221.

54 Bogue J, Power K. Suicide in Scottish Prisons, 1976-93. *J Forensic Psychiatry* 1995; **6**: 527–40.

55 Crighton D, Towl G. Self–inflicted deaths in prison in England and Wales: An analysis of the data for 1988–90 and 1994–95. *Issues Criminol Leg Psychol* 1997.

56 Scott-Denoon K. BC Corrections: A study of suicides 1970-1980. *Br Columbia Correct Branch* 1984.

57 Dooley E. Prison suicide in England and Wales, 1972–87. *Br J Psychiatry* 1990; **156**: 40–45.

58 DuRand CJ, Burtka GJ, Federman EJ, Haycox JA, Smith JW. A quarter century of suicide in a major urban jail: Implications for community psychiatry. *Am J Psychiatry* 1995; **152**: 1077–80.

59 Frickey RC. Suicide in the US Federal Prison System. Uniformed Services Univ of The Health Sciences Bethesda, 1999.

60 Fruehwald S, Frottier P, Eher R, Gutierrez K, Ritter K. Prison suicides in Austria, 1975-1997. *Suicide Life Threat Behav* 2000; **30**: 360–9.

61 Hatty S, Walker JR. A national study of deaths in Australian prisons. Australian Institute of Criminology Canberra, 1986.

62 Hayes LM. National Study of Jail Suicides: Seven Years Later. *Psychiatr Q* 1989; **60**: 7–29.

63 Hurley W. Suicides by prisoners. *Med J Aust* 1989; **151**: 188–190.

64 Kovasznay B, Miraglia R, Beer R, Way B. Reducing suicides in New York State correctional facilities. *Psychiatr Q* 2004; **75**: 61–70.

65 Laishes J. Inmate suicides in the Correctional Service of Canada. *Crisis* 1997; **18**: 157–162.

66 Epidemiology of Suicides in New York State Correctional Facilities. New York, NY: US Medical Review Board, 1998.

67 Suicide in Prison. London, United Kingdom: National Association for the Care and Resettlement of Offenders, 1990.

68 Self-Inflicted Deaths in Custody: Six-Year Over- view: 1999/2000 to 2004/2005 [internal report]. London, United Kingdom: National Offender Management Service, 2005.

69 Suicide Statistics on Sexual Offenders [from a database of self-inflicted deaths in England and Wales 1999/2000 to 2003/2004]. London, United Kingdom: National Offender Management Service, 2007.

70 Salive ME, Smith GS, Brewer TF. Suicide mortality in the Maryland state prison system, 1979 through 1987. *JAMA* 1989; **262**: 365–369.

71 Snow L, Paton J, Oram C, Teers R. Self-inflicted deaths during 2001: an analysis of trends. *Br J Forensic Pract* 2002; **4**.

72 Tatarelli R, Mancinelli I, Taggi F, Polidori G. Suicide in Italian prisons in 1996 and 1997: A descriptive epidemiological study. *Int J Offender Ther Comp Criminol* 1999; **43**: 438–447.

73 Towl GJ, Crighton DA. Suicide in prisons in England and Wales from 1988 to 1995. *Crim Behav Ment Health* 1998; **8**: 184–192.

74 White TW, Schimmel DJ, Frickey R. A comprehensive analysis of suicide in federal prisons: a fifteen-year review. *J Correct Health Care* 2002; **9**: 321–343.

75 He XY, Felthous AR, Rd HC, Nathan P, Veasey S. Factors in prison suicide: one year study in Texas. *J Forensic Sci* 2001; **46**: 896–901.

76 Skegg K, Cox B. Suicide in custody: occurrence in Maori and nonMaori New Zealanders. *N Z Med J* 1993; **106**: 1–3.

77 Novick LF, Remmlinger E. A study of 128 deaths in New York City correctional facilities (1971-1976): implications for prisoner health care. *Med Care* 1978; **16**: 749–756.

78 Noonan M. Mortality in State Prisons in U.S, 2001-2014. Department of Justice, 2016.

79 Noonan M. Mortality in Local Jails in U.S, 2000-2014. Department of Justice, 2016.
